# Minimal-Invasive Versus Open Hepatectomy for Colorectal Liver Metastases: Bicentric Analysis of Postoperative Outcomes and Long-Term Survival Using Propensity Score Matching Analysis

**DOI:** 10.3390/jcm9124027

**Published:** 2020-12-13

**Authors:** Sebastian Knitter, Andreas Andreou, Daniel Kradolfer, Anika Sophie Beierle, Sina Pesthy, Anne-Christine Eichelberg, Anika Kästner, Linda Feldbrügge, Felix Krenzien, Mareike Schulz, Vanessa Banz, Anja Lachenmayer, Matthias Biebl, Wenzel Schöning, Daniel Candinas, Johann Pratschke, Guido Beldi, Moritz Schmelzle

**Affiliations:** 1 Department of Surgery, Campus Charité Mitte and Campus Virchow Klinikum, Chariteé-Universitätsmedizin Berlin, Corporate Member of Freie Universität Berlin, Humboldt-Universität zu Berlin, and Berlin Institute of Health, 13353 Berlin, Germany; sebastian.knitter@charite.de (S.K.); anika.beierle@charite.de (A.S.B.); sina.pesthy@charite.de (S.P.); anne-christine.eichelberg@charite.de (A.-C.E.); anika.kaestner@charite.de (A.K.); linda.feldbruegge@charite.de (L.F.); felix.krenzien@charite.de (F.K.); mareike.schulz@charite.de (M.S.); matthias.biebl@charite.de (M.B.); wenzel.schoening@charite.de (W.S.); johann.pratschke@charite.de (J.P.); 2 Department of Visceral Surgery and Medicine, lnselspital, Bern University Hospital, University of Bern, 3010 Bern, Switzerland; andreas.andreou@insel.ch (A.A.); daniel.kradolfer@insel.ch (D.K.); Vanessa.BanzWuethrich@insel.ch (V.B.); Anja.Lachenmayer@insel.ch (A.L.); daniel.candinas@insel.ch (D.C.)

**Keywords:** colorectal liver metastases, laparoscopic liver surgery, minimal invasive surgery

## Abstract

Minimal-invasive hepatectomy (MIH) has been increasingly performed for benign and malignant liver lesions with most promising short-term results. However, the oncological role of MIH in the treatment of patients with colorectal liver metastases (CRLM) needs further investigation. Clinicopathological data of patients who underwent liver resection for CRLM between 2012 and 2017 at the Department of Surgery, Charité-Universitätsmedizin Berlin, and the Inselspital Bern were assessed. Postoperative outcomes und long-term survivals of patients following MIH were compared with those after conventional open hepatectomy (OH) after 1:1 propensity score matching. During the study period, 229 and 91 patients underwent liver resection for CRLM at the Charité Berlin and the Inselspital Bern, respectively. Patients who underwent MIH in one of the two centers (*n* = 69) were compared with a matched cohort of patients who underwent OH. MIH was associated with lower complication rates (23% vs. 44%, *p* = 0.011), shorter length of intensive care unit stay (ICU, 1 vs. 2 days, *p* = 0.043), shorter length of hospital stay (7 vs. 11 days, *p* < 0.0001), and a reduced need for intraoperative transfusions (12% vs. 25%, *p* = 0.047) compared to OH. R0 status was achieved in 93% and 75% of patients after MIH and OH, respectively (*p* = 0.005). After a median follow-up of 31 months, MIH resulted in similar five-year overall survival (OS) rate (56% vs. 48%, *p* = 0.116) in comparison to OH. MIH for CRLM is associated with lower postoperative morbidity, shorter length of ICU and hospital stay, reduced need for transfusions, and comparable oncologic outcomes compared to the established OH. Our findings suggest that MIH should be considered as the preferred method for the treatment of curatively resectable CRLM.

## 1. Introduction

The most common site of metastatic tumor spread in patients with colorectal cancer is the liver. At time of diagnosis, 20% of patients present with synchronous colorectal liver metastases (CRLM) [1], and up to 25% of patients are at risk to develop metachronous CRLM over time [2]. Liver resection remains the mainstay of a multimodal curative therapeutic approach for CRLM, enabling long-term survivals. In the last decades, advances in the multimodal treatment of patients with CRLM have achieved five-year overall survival rates of up to 58% after hepatectomy [3,4].

Since the introduction of laparoscopic liver surgery in the early 1990s [5,6], minimal-invasive hepatectomy (MIH) has been widely adopted for benign and malignant lesions of the liver, including hepatocellular carcinoma [7,8] and CRLM [9]. Doubts about laparoscopic liver resection concerning the general complexity of the technique and cost-effectiveness are gradually declining, since benefits in short-term outcomes have been reported in several retrospective studies and were confirmed in meta-analyses [10,11]. MIH for CRLM has been associated with less intraoperative blood loss, lower need for transfusion, lower postoperative morbidity rates, and shorter length of hospital stay in comparison to open hepatectomy (OH) [10,12,13]. Reports on oncologic results have already demonstrated the non-inferiority of MIH in terms of the rate of patients with tumor-free resection margins (R0 resection), overall survival (OS), and disease-free survival (DFS) [9,14]. These findings have indicated that MIH is a valid alternative to OH in the most recent Southampton guidelines for laparoscopic liver surgery [15].

Scientific evidence on the feasibility of MIH for CRLM is mostly based on retrospectively collected data. To date, only one randomized controlled study (RCT) from Norway has been conducted showing benefits for minimal-invasive CRLM resection concerning postoperative morbidity and length of hospital stay [13]. However, this study was single-center and included only patients with minor resections, thus may not be widely applicable for patients with extended disease.

Although long known [16], propensity-score matching (PSM) analysis gained popularity in recent years as a statistical method to adjust for known confounding factors and thus reduce the impact of selection bias in retrospective studies [17]. PSM has been often used for the comparison of surgical techniques in an effort to create comparable treatment groups [10]. Using propensity-score based analysis, previous European multi-center studies have given insight into the short- and long-term outcomes of MIH in comparison to OH for CRLM [18,19]. In this study, we aimed to share the current experience of two major hepatobiliary centers that regularly perform MIH.

The objective of this study was to evaluate the postoperative and oncologic outcomes of patients undergoing MIH for CRLM compared to those of patients undergoing OH in a bi-centric setting using PSM analysis. In addition, the inter-center comparison of postoperative and long-term outcomes between two major hepatobiliary centers in Berlin, Germany, and Bern, Switzerland, intended to provide further insight into each center’s approach to the treatment of patients with CRLM.

## 2. Experimental Section

### 2.1. Patient Inclusion Criteria

Approval for this study was obtained from the Ethics Commission of Charité-Universitätsmedizin Berlin (EA2/006/16) and the Ethics Commission of the Canton of Bern (2018-01576). Clinicopathological data on 229 and 91 consecutive patients who underwent resection for CRLM from 2012 to 2017, at the Department of Surgery, Campus Charité Mitte and Campus Virchow Klinikum, Charité-Universitätsmedizin Berlin, and the Department of Visceral Surgery and Medicine, lnselspital, Bern University Hospital, University of Bern, respectively, were collected.

Inclusion criterion was a curative intended resection, defined as the ability to remove all radiologically evident disease. Patients were excluded from the analysis if they were <18 years old, if extended hemihepatectomies were performed, and if microwave ablations or other surgical procedures (e.g., resection of the primary tumor) were concomitantly performed with hepatectomy.

### 2.2. Preoperative Assessment

Patients with CRLM presenting in each department routinely underwent a standardized preoperative evaluation protocol that included medical history, physical examination, serum laboratory tests, and an anesthesia evaluation. Tumor staging and the estimation of the future liver remnant (FLR) volume was determined via cross-sectional imaging (triphasic contrast-enhanced computed tomography or magnetic resonance imaging with liver-specific contrast agents) as needed.

An institutional multidisciplinary tumor board in each center consisting of surgeons, hepatologists, oncologists, and specialized radiologists discussed each case and recommended the best individual treatment strategy for each patient.

### 2.3. Surgical Procedure

Surgical procedures were performed as previously described [20,21,22,23]. At the beginning of every procedure, following laparotomy or laparoscopy, the peritoneal cavity was examined to rule out any previously undiagnosed tumor spread. Intraoperative ultrasound of the liver was used to validate the exact location and extent of CRLM as determined by imaging studies. Conventional OH was initiated with a modified Makuuchi incision [24]. For MIH in both centers, patients were kept in a supine position with legs spread apart (French position) [25]. MIH was performed either via multiport laparoscopic surgery (MLS, transumbilical 12 mm optical trocar and further 5 mm and 12 mm trocars), single-incision laparoscopic surgery (SILS; GelPort^®^, Applied Medical, Rancho Santa Margarita, CA, USA, via 4–5 cm midline incision for three trocars, additional 5 mm port in upper left abdominal quadrant, if needed), or hand-assisted laparoscopic surgery (HALS; handport via 5 cm supraumbilical incision and 2–3 additional 12 mm ports). Specimen were retrieved via a Pfannenstiel incision, extension of the umbilical incision or by using an existing scar. Total or selective hepatic vascular exclusion was utilized for major parenchymal transections, as needed. For MIH in both centers, liver parenchyma dissection was performed using a combination of following devices and instruments: energy devices (Thunderbeat^®^, Olympus K.K., Tokyo, Japan, or Harmonic Ace^®^, Ethicon Inc., Somerville, NJ, USA), laparoscopic cavitron ultrasonic surgical aspirator (CUSA^®^, Valleylab Boulder, CO, USA), Waterjet (ERBEJET^®^, ERBE Tübingen, Germany), and vascular stapler (Echelon™, Ethicon, Somerville, NJ, USA) or Endo GIA™ (Medtronic, Dublin, Ireland).

The resection of ≥3 contiguous liver segments defined major hepatectomy according to Couinaud’s classification [26]. Location of CRLM was stratified by technical difficulty in segments II, III, IVb, V, and VI, and segments I, IVa, VII, and VIII. The latter was defined as technical more difficult.

### 2.4. Postoperative Management

After surgery, patients were occasionally admitted to a surgical intensive care unit (ICU) if needed, where they were monitored for postoperative complications such as intra-abdominal bleeding, infection, biliary fistula, wound infection, pneumonia, pleural effusions, and liver failure. After routine removal of the nasogastric tube on the same day, oral intake and mobilization was anticipated on postoperative day 1. Intra-abdominal drains were either not used at all or removed as soon as the discharge was unremarkable. Blood tests were postoperatively regularly performed to assess liver function and cholestasis and rule out the development of an infectious collection. Any complication or death within 90 days after surgery defined postoperative morbidity and mortality, respectively. Postoperative complications were graded according to the classification of Clavien and Dindo and major complications were defined as ≥3a [27]. After surgery, all cases were re-evaluated in our multidisciplinary tumor board and postoperative treatment was recommended according to current guidelines [28].

### 2.5. Histological Evaluation

Resected specimens were evaluated by a pathologist to confirm the diagnosis of CRLM and to determine the resection margin status (R). R0 was defined as a surgical margin of ≥1 mm free of malignant cells [29].

### 2.6. Statistical Analysis

Propensity score analysis was used to match patients who underwent MIH for CRLM with a cohort of patients who were treated with OH in each center separately. A 1:1 PSM was performed using a logistic regression model with a match tolerance of 0.1 based on the following matching parameters: patient age, sex, ASA (American Society of Anesthesiologists) status, comorbidities (diabetes, hypertension, coronary heart disease, pulmonary disease, and/or renal disease), presence of solitary or multiple CRLM, sequence of hepatic tumor spread (synchronous or metachronous), preoperative chemotherapy, and resection extent (including major or minor hepatectomy). Matched cohorts from both centers were then merged for a pooled comparison of MIH with OH.

Quantitative and qualitative variables were expressed as medians (range) and frequencies. The chi-square or Fisher’s exact test for categorical variables, and the Wilcoxon signed-rank test for continuous variables, were used as appropriate to compare between groups. Patient characteristics and postoperative outcomes were compared between the matched MIH and OH cohorts as well as between the MIH patients in Berlin and Bern.

Using the Kaplan-Meier method, overall survival (OS) was calculated from the date of resection to the date of death or last follow-up and disease-free survival (DFS) was calculated from the date of resection to the date of first recurrence or last follow-up. Comparisons between survival rates were performed using log-rank tests.

*p* values < 0.05 were considered statistically significant. Statistical analyses were performed using the SPSS software package, version 25 (IBM, Armonk, NY, USA).

## 3. Results

### 3.1. Patient Characteristics

During the study period, 229 and 91 consecutive patients underwent hepatectomy for CRLM at the Department of Surgery, Campus Charité Mitte and Campus Virchow-Klinikum, Charité Berlin, and the Department of Visceral Surgery und Medicine, lnselspital, Bern, respectively, and were included in this study. OH was performed in 251 patients (78%) (*n* = 185 in Berlin, *n* = 66 in Bern) and 69 patients (22%) were treated with MIH (*n* = 44 in Berlin, *n* = 25 in Bern).

Patient cohorts in Berlin differed significantly before matching (MIH vs. OH) regarding the rate of patients presenting with CRLM > 50 mm including 35% and 16% in the OH and MIH group, respectively (*p* = 0.016). Additionally, the extent of liver resection was significantly different between the two groups (*p* < 0.0001), since there were more open major hepatectomies during the study period. Before matching MIH with OH in Bern, there were significant differences between the two groups regarding the proportion of patients presenting with single CRLM (76% vs. 42%, *p* = 0.003) and CRLM > 50 mm (12% vs. 36%, *p* = 0.023). In addition, the N stage of the primary tumor was more advanced in the OH group (*p* = 0.048). Significantly less major hepatectomies (4% vs. 33%, *p* = 0.004) and less anatomic resections (4% vs. 55%, *p* < 0.0001) were performed in the MIH cohort compared to the OH group.

After PSM, pooled analysis of MIH (*n* = 69 patients) versus OH (*n* = 69 patients) is summarized in Table 1. Both groups (MIH vs. OH) were comparable regarding sex (male: 68% vs. 55%, *p* = 0.115), median age (65 vs. 63 years, *p* = 0.210), median BMI (25 vs. 25 kg/m^2^, *p* = 0.718), ASA physical status (*p* = 0.470), and location of the primary tumor (colon and rectum: 54% vs. 61% and 46% vs. 39%, *p* = 0.390). Moreover, no significant differences regarding the sequence of liver tumor spread (synchronous: 46% vs. 45%, *p* = 0.864), and the proportion of patients presenting with CRLM > 50 mm (15% vs. 27%, *p* = 0.074) or solitary CRLM (51% vs. 44%, *p* = 0.439) were found. However, CRLM were more frequently located in segments I, IVa, VII, and VIII in the OH group (*p* = 0.005). The rate of patients treated with preoperative chemotherapy was comparable between both groups (60% vs. 62%, *p* = 0.727). Anatomic resections were performed in 39% and 51% in the MIH and OH group, respectively (*p* = 0.171). Both groups had equal amounts of major resections (33% vs. 33%, *p* = 1), and the extent of hepatectomy was comparable (*p* = 0.660) between the two groups. The median duration of surgery was similar between the groups (218 vs. 250 min, *p* = 0.078). Positive resections margins were found in 7% and 25% after MIH and OH, respectively (*p* = 0.005). A comparable number of patients were monitored on the ICU postoperatively (83% vs. 88%, *p* = 0.370). The length of ICU stay (1 vs. 2 days, *p* = 0.043) and length of hospital stay (7 vs. 11 days, *p* < 0.0001) were significantly shorter after MIH than after OH. The need for blood transfusions was reduced after MIH (12% vs. 25%, *p* = 0.047).

### 3.2. Postoperative Morbidity and Mortality

Postoperative morbidity was significantly lower after MIH than after OH (23% vs. 44%, *p* = 0.011). Postoperative mortality was not significantly different between the two techniques (1% vs. 3%, *p* = 0.559).

### 3.3. Overall Survival and Disease-Free Survival

**Figure 1:**
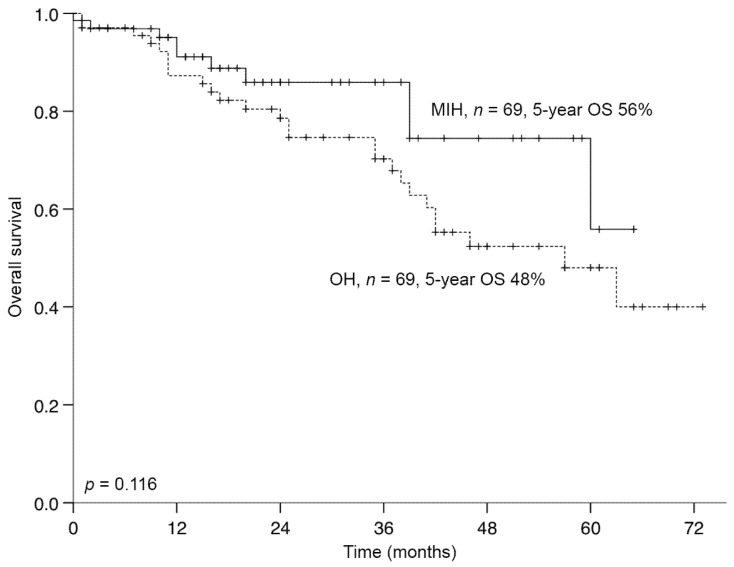
Overall survival of propensity-score matched patients who underwent hepatectomy for CRLM in Berlin and Bern (*n* = 138). MIH, minimal-invasive hepatectomy; OS, overall survival; OH, open hepatectomy; CRLM, colorectal liver metastases

After a median follow-up time of 31 months, the 5-year OS rates were 56% and 48% after MIH and OH, respectively (*p* = 0.116; Figure 1). Five-year DFS rates were 46% and 27% after MIH and OH, respectively (*p* = 0.018).

### 3.4. Comparison of Clinicopathological Characteristics and Outcomes of Patients Who Underwent MIH in Berlin vs. Bern

Characteristics of patients who underwent MIH in Berlin (*n* = 44) were compared with those of patients in Bern (*n* = 25) (Table 2). Patient-related parameters were comparable between the two centers. No significant differences were found concerning age (*p* = 0.453), BMI (*p* = 0.513), ASA status (*p* = 0.163), or presence of comorbidities. The rate of patients with CRLM > 50 mm (16% vs. 12%, *p* = 0.657) and synchronous CRLM (43% vs. 52%, *p* = 0.480) was equivalent between the two centers. CRLM were equally located in segments II, III, IVb, V, and VI (*p* = 0.180), and segments I, IVa, VII, and VIII of the liver (*p* = 0.306). However, patients presented with solitary CRLM more frequently in Bern than in Berlin (76% vs. 36%, *p* = 0.002). More major (50% vs. 4%, *p* < 0.0001) and anatomic resections (59% vs. 4%, *p* < 0.0001) were performed in Berlin than in Bern. In general, the extent of MIH was different between Berlin and Bern (*p* = 0.002). Whereas a large part of minimal-invasive resections in Berlin were hemihepatectomies, the majority of laparoscopic treated patients in Bern underwent wedge resections or segmentectomies, thus also resulting in significant differences regarding the median duration of the operation (290 vs. 125 min, *p* < 0.0001), postoperative morbidity rates (32% vs. 8%, *p* = 0.024), and length of hospital stay (9 vs. 5 days, *p* < 0.0001). There were more patients in intensive care in Berlin than in Bern (91% vs. 68%, *p* = 0.022); however, length of ICU stay was comparable between the centers (1 vs. 2 days, *p* = 0.283). Postoperative mortality after MIH was comparable in both centers (*p* = 1). Additionally, there were no significant differences regarding positive resection margins (*p* = 0.151), and the need for transfusions (*p* = 1).

**Figure 2:**
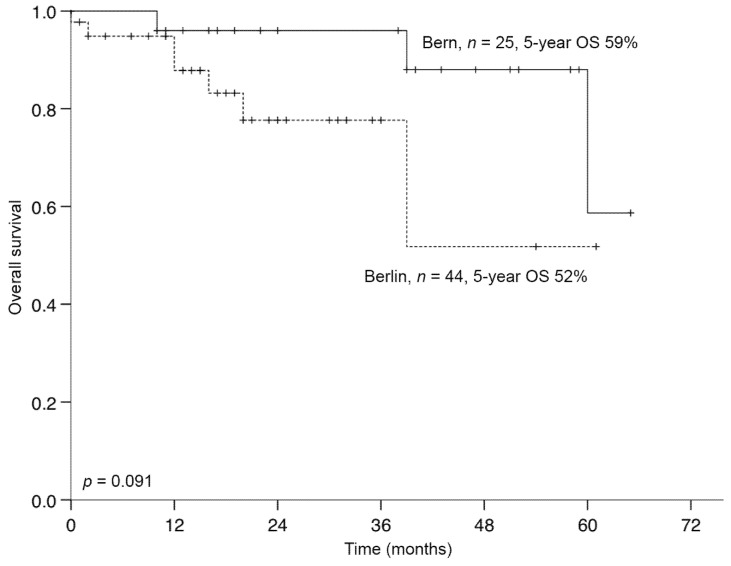
Overall survival of patients who underwent minimal-invasive hepatectomy for CRLM in Berlin or Bern (*n* = 69). OS, overall survival; CRLM, colorectal liver metastases

Five-year OS rates (Berlin vs. Bern: 52% vs. 59%, *p* = 0.091; Figure 2) and DFS rates (Berlin vs. Bern: 47% vs. 44%, *p* = 0.577) were statistically equivalent between the two centers.

Forty-three percent (*n* = 3/7) and 67% (*n* = 2/3) of patients who deceased following MIH in the observed time period in Berlin and Bern, respectively (*p* = 1), died due to cancer progression including intrahepatic recurrence. Two of the three patients suffering cancer-related death in Berlin had initially underwent major MIH because of extended liver tumor burden, and none of these three patients were selected for repeat hepatectomy due to extended recurrent disease burden or insufficient FLR. Instead of re-resection, one patient underwent local brachytherapy, one patient was administered palliative chemotherapy, and the third was lost to follow-up. In Bern, both patients, who died of cancer progression following laparoscopic hepatectomy underwent initially minor liver resections (bisegmentectomy and wedge resection) and were able to undergo repeat MIH for limited intrahepatic recurrence. After diagnosis of intrahepatic recurrence, patients lived for another 6, 9, and 10 months in Berlin, and for another 27 and 28 months in Bern, respectively.

## 4. Discussion

Our study on a bicentric experience from the Charité Berlin (Campus Charité Mitte and Campus Virchow-Klinikum) and the Inselspital Bern compared the postoperative and oncologic outcomes of patients who underwent MIH for CRLM with those of propensity-score matched patients treated with OH. In comparison to conventional OH, our results indicated lower postoperative complication rates, shorter length of ICU and hospital stay, and lower rates of intraoperative blood transfusions for patients undergoing MIH. MIH was associated with oncologic outcomes equivalent to those after OH.

The advantages of MIH over OH for CRLM have been previously reported in the literature. Recent studies found benefits regarding postoperative morbidity, length of hospital stay, blood loss, and the need for transfusions with equivalent oncologic outcomes in comparison to OH [10,12,14,30,31,32]. These findings have been mainly derived from retrospective data since RCTs are often faced with methodical and patient-recruitment related challenges [33]. So far, only one RCT has been completed evaluating MIH for CRLM [13] while another had to be cancelled early due to a slow accrual process [34]. For this reason, PSM analysis has been introduced aiming to overcome treatment bias in retrospective studies by assembling patient cohorts with minimal differences in clinicopathological features allowing for a meaningful comparison [35,36]. To date, consensus regarding the optimal parameters that should be included in PSM is lacking. Since our objective was to eliminate all patient- and tumor-related factors that could influence outcomes after surgery, we decided to include all variables concerning the general condition of the patient, the tumor characteristics, the administration of preoperative chemotherapy, and the extent of hepatectomy. Various studies performed PSM to compare the outcomes of laparoscopic versus open resection for CRLM, most recently coming from Italy [12], and the USA [14]. Earlier studies were analyzed in a meta-analysis which is entirely based on propensity-score matched data [10].

Within the framework of our PSM analysis, we report on a comparably high rate of major MIH for CRLM with 50% in Berlin and 33% in the entire cohort, respectively [12]. Of note, Fretland et al. defined parenchymal-sparing liver resections in the design of their RCT with the switch to major hepatectomy if needed [13]. To our knowledge, only few groups performed >50% major hepatectomies in the laparoscopic group [31,37]. Due to the influence of the extent of hepatectomy on postoperative outcomes, these findings need to be acknowledged when comparing the results of our study with others. However, our study confirmed the findings of previous studies, which have shown that MIH is associated with lower morbidity after surgery compared to OH [13,14,32]. Postoperative morbidity (23% vs. 44%, *p* = 0.011) was significantly reduced after MIH, whereas major postoperative morbidity was comparable between MIH and OH (17% vs. 25%, *p* = 0.296).

Moreover, our results showed at least equivalent oncologic outcomes between MIH and OH (5-year OS: 56% vs. 48%, *p* = 0.116; 5-year DFS: 46% vs. 27%, *p* = 0.018). Previous studies reported comparable survivals after open and laparoscopic hepatectomy for CRLM [12]. Patient- and tumor-related factors that may have an influence on survival after surgery are comorbidities, size, and number of CRLM, rate of preoperative chemotherapy, and extent of hepatectomy. These parameters were comparable between the groups after PSM, supporting our opinion that MIH may provide oncologically sufficient outcomes.

Furthermore, oncologic outcomes are generally known to be influenced by the presence of malignant cells in the surgical margins (R1) allowing for early tumor recurrence [29]. Notably, positive resection margins were significantly decreased after MIH in comparison to OH in this study (7% vs. 25%, *p* = 0.005). However, median resection margin width in R0 resected patients was comparable between OH and MIH, as previously shown [38]. Margin width following MIH was also comparable between the two centers. The implementation of standardized and routinely performed intraoperative ultrasound both in MIH and OH has allowed for higher rates of R0 resections in the recent years. However, the possibility that the surgeon might evaluate and determine the resection margins more conservatively when performing MIH [9], especially due to the different tactile feedback and during the learning curve could explain the improved R0 resection rate after MIH. Additionally, despite thorough PSM taking into consideration numerous cancer-related characteristics we could probably not entirely eliminate bias in the selection of the matched OH cases resulting in the inclusion of some more advanced disease. Furthermore, CRLM were more frequently located in segments I, IVa, VII, and VIII of the liver in the OH group compared to the MIH group. Hepatectomy on these segments are known to be more difficult to perform [39], and this may have translated into a higher R1 rate in this group, and may have negatively influenced the oncologic outcome among OH patients [40]. In addition, more extensive tumor burden requiring OH may also be reflective of unfavorable tumor biology, which, in turn, may be responsible for higher R1 resection rates [41,42,43].

Currently, perioperative chemotherapy has been widely adapted as an integral component in the multimodal treatment of patients with CRLM [44]. The establishment of modern chemotherapy regimens was one reason to exclude patients treated before 2012. Guidelines recommend the use of preoperative systemic therapy to downsize the hepatic tumor burden and consequently convert patients with unresectable disease to a resectable state [45,46]. Additionally, patients with initially resectable CRLM may profit from preoperatively administered chemotherapy by reducing the presence of malignant cells in the resection margins resulting in prolonged progression-free survival [44]. Our data suggest that patients in both cohorts (MIH and OH) may have benefited equally from the positive effects of chemotherapy as it was administered to equivalent percentages of patients preoperatively. However, possible side effects of neoadjuvant cytotoxic agents (e.g., steatosis, steatohepatitis, or sinusoidal changes) need to be taken into consideration, impairing the function and regenerative capacity of the otherwise healthy remaining liver tissue [47].

Finally, in an effort to elucidate the improved OS for patients after MIH, postoperative morbidity following liver resection for CRLM may have played a role as it has been proven to affect OS and DFS negatively [48,49,50,51,52]. It is hypothesized that postoperative morbidity prolongs a phase of immunosuppression allowing residual tumor cells to proliferate and to induce local recurrence [53]. We found a significantly reduced incidence of overall complications after MIH in comparison to OH in our study, suggesting a positive impact on long-term survival after MIH. Moreover, postoperative complications may have delayed the onset of adjuvant chemotherapy as part of the multimodal treatment concept further contributing to diminished overall survival, as seen in patients after colorectal cancer surgery [54].

Another interesting finding of our study was that MIH correlated with a shorter length of ICU and hospital stay compared to OH. Laparoscopic techniques are considered to diminish the stress that surgical procedures exert on the human body, and thus help to preserve and eventually restore organ function after surgery in an accelerated timeline. Benefits of MIH include earlier postoperative oral intake, optimized postoperative pain control, and earlier mobilization after surgery. In addition to the reduced morbidity, these factors may have also contributed to an earlier discharge for patients after MIH.

In this study, we compared the results of patients, who underwent MIH, in the Charité Berlin und the Inselspital Bern. Both groups were equivalent regarding patient-related characteristics but differed significantly in tumor-specific features. In Bern, MIH was mostly selected for patients with solitary CRLM, and segmental resections were predominantly performed. This resulted in reduced morbidity (8% vs. 32%, *p* = 0.024) and shorter length of hospital stay after MIH in comparison to Berlin (5 vs. 9 days, *p* < 0.0001) without diminishing R0 resection rates. In contrast, more patients in Berlin presented with higher tumor burden making more extensive hepatic resections necessary. These major MIHs for multiple CRLM were then followed by higher complication rates and longer length of hospital stay in comparison to Bern. However, the results from both centers showed comparable low mortality rates, and prolonged long-term survivals.

When deciding for a liver resection strategy, surgeons are confronted with two conflicting objectives; on the one side, extensive hepatic disease requires an adequate resection extent in order to achieve R0 resection and prevent tumor recurrence. On the other side, as much non-tumorous liver tissue as possible needs to be preserved aiming to avoid postoperative liver insufficiency. This is further aggravated by the aforementioned risks of preoperatively administered chemotherapy on the histopathological and functional integrity of the residual liver parenchyma. An initially major hepatectomy may impede the selection of patients for repeat liver resection in case of intrahepatic recurrence. Additionally, extended tumor burden requiring hemihepatectomy or more is a surrogate factor for advanced disease with a higher risk for earlier and disseminated recurrence. In our study, patients who died of intrahepatic recurrence following MIH in Berlin, presented with high tumor burden in the first place making extended hepatectomy necessary. Repeat hepatectomy for recurrence could not be offered and these patients died within the first year after recurrence. In contrast, patients who died of intrahepatic recurrence following minor MIH in Bern could undergo repeat parenchymal-sparing liver resection for single CRLM and lived for another two years. The fact that more patients in Berlin than in Bern died without recurrence from non-cancer related causes, could be one reason for the higher DFS but lower OS in Berlin (not statistically significant differences).

In the current literature, the optimal extent of hepatic resection remains unclear. Often, no differences were found between parenchymal-sparing and anatomic resections [55,56], but the current trend moves towards parenchymal-sparing resections [57]. In this regard, CRLM needs to be acknowledged as a chronic disease. By limiting hepatectomies to parenchymal-sparing resections whenever possible, re-resections for recurrent CRLM are made possible [58]. Interestingly, improved oncologic outcomes were not associated with an increase in margin width for R0 resections [59] leading to the recommendation in the current Southampton guidelines that parenchymal-sparing resections should be preferably conducted for patients with CRLM [15], which was implemented in both centers if this was allowed by the extent of the tumor burden. Nevertheless, prolonged OS could be achieved in both centers in our study, and especially DFS was not compromised by parenchymal-sparing resections. In summary, despite of favorable short-term and comparable long-term outcomes after MIH found in this study, the decision for either MIH or OH should be individually made for each patient and should be based on patient- and tumor-related factors.

Our present retrospective study has also several limitations. Firstly, conclusions should be carefully drawn due to the rather small cohorts and the retrospective nature of data collection. We tried to challenge this issue by establishing a bi-centric cohort pooling data from two specialized centers with large experience in hepatobiliary surgery. Surgeon- and center-related bias have been eliminated by matching MIH with OH for each center separately. Thus, we could assure that same number of OH and MIH were included from each center, respectively. In addition, it was our objective to create a meaningful statistical comparison by performing PSM to eliminate known covariates. Nevertheless, unused and especially unknown confounders could have influenced our results. Of note, the investigation of somatic gene mutations gains increasingly importance in the treatment of CRLM [60,61,62]; however, this was not within the scope of this study. The introduction of laparoscopic surgery for CRLM during the study period is associated with a possible learning curve, which may have influenced the outcomes [63,64]. However, standardized laparoscopic procedures performed by experienced hepatobiliary surgeons in both high-volume centers resulted in favorable short- and long-term outcomes underling the advantages of MIH for CRLM.

## 5. Conclusions

MIH for CRLM was associated with lower overall postoperative morbidity, and shorter length of ICU and hospital stay compared to OH. Oncologic outcomes after MIH were at least equivalent compared to those after OH. Therefore, our results support our current approach that MIH should be preferred for patients presenting with resectable CRLM.

## Figures and Tables

**Table 1:** Characteristics of propensity-score matched patients who underwent hepatectomy for CRLM in Berlin and Bern (*n* = 138).

Variable	OH (*n* = 69)	MIH (*n* = 69)	*p*
Gender, *n* (%)			0.115
Female	31 (45)	22 (32)	
Male	38 (55)	47 (68)	
Age, years, median (range)	63 (30–86)	65 (27–89)	0.210
Age > 65 years, *n* (%)	32 (46)	35 (51)	0.609
BMI, kg/m^2^, median (range)	25 (18–46)	25 (18–40)	0.718
BMI > 30 kg/m^2^, *n* (%)	11 (16)	14 (21)	0.480
ASA physical status, *n* (%)			0.470
1	2 (3)	0 (0)	
2	34 (49)	32 (46)	
3	32 (47)	35 (51)	
4	1 (1)	2 (3)	
Comorbidities, *n* (%)			
Diabetes	10 (15)	12 (17)	0.642
Hypertension	29 (42)	29 (42)	1
Coronary heart disease	4 (6)	7 (10)	0.346
Pulmonary disease	4 (6)	4 (6)	1
Renal disease	4 (6)	6 (9)	0.511
Localization of primary, *n* (%)			0.390
Colon	42 (61)	37 (54)	
Rectum	27 (39)	32 (46)	
Synchronous CRLM, *n* (%)	31 (45)	32 (46)	0.864
Size of biggest CRLM > 50 mm, *n* (%)	18 (27)	10 (15)	0.074
Solitary CRLM, *n* (%)	30 (44)	35 (51)	0.439
CRLM in segments II, III, IVb, V, and VI, *n* (%)	56 (81)	63 (91)	0.084
CRLM in segments I, IVa, VII, and VIII, *n* (%)	52 (75)	36 (52)	0.005
Preoperative chemotherapy, *n* (%)	43 (62)	41 (60)	0.727
T stage of primary, *n* (%)			0.360
1	4 (6)	3 (5)	
2	4 (6)	8 (13)	
3	43 (64)	41 (67)	
4	16 (24)	9 (15)	
N stage of primary, *n* (%)			0.038
0	23 (34)	33 (54)	
1	33 (49)	16 (26)	
2	11 (16)	11 (18)	
3	0 (0)	1 (2)	
UICC stage of primary, *n* (%)			0.031
1	5 (7)	7 (11)	
2	7 (10)	14 (21)	
3	23 (34)	9 (14)	
4	33 (49)	36 (54)	
Tumor grading of primary, *n* (%)			0.443
G1	2 (4)	0 (0)	
G2	47 (82)	38 (84)	
G3	8 (14)	7 (16)	
Postoperative ICU stay, *n* (%)	59 (88)	57 (83)	0.370
Length of ICU stay, days, median (range)	2 (0–37)	1 (0–23)	0.043
Length of hospital stay, days, median (range)	11 (4–109)	7 (2–59)	<0.0001
90-day complications, *n* (%)	30 (44)	16 (23)	0.011
90-day major complications, *n* (%)	17 (25)	12 (17)	0.296
90-day mortality, *n* (%)	2 (3)	1 (1)	0.559
Anatomic resection, *n* (%)	35 (51)	27 (39)	0.171
Major resection, *n* (%)	23 (33)	23 (33)	1
Positive resection margins, *n* (%)	17 (25)	5 (7)	0.005
Resection margin width in R0 resected patients, mm, median (range)	2 (1–20)	3 (1–40)	0.430
Surgical technique, *n* (%)			0.660
right hepatectomy	13 (19)	12 (17)	
right hepatectomy and wedge resections or segmental resections	6 (9)	8 (12)	
left hepatectomy	4 (6)	1 (1)	
left hepatectomy and wedge resections or segmental resections	0 (0)	2 (3)	
left lateral hepatectomy	4 (6)	4 (6)	
segmentectomy/wedge resection	30 (43)	30 (43)	
bisegmentectomy	12 (17)	12 (17)	
Need for transfusion, *n* (%)	17 (25)	8 (12)	0.047
Duration of operation, minutes, median (range)	250 (106–513)	218 (46–602)	0.078
Postoperative chemotherapy, *n* (%)	22 (32)	22 (32)	1

CRLM, colorectal liver metastases; OH, open hepatectomy; MIH, minimal-invasive hepatectomy; BMI, body mass index; ASA, American Society of Anesthesiologists; UICC, Union internationale contre le cancer; ICU, intensive care unit.

**Table 2:** Comparison of characteristics and outcomes between patients who underwent minimal-invasive hepatectomy for CRLM in Berlin or Bern (*n* = 69).

Variable	Berlin (*n* = 44)	Bern (*n* = 25)	*p*
Gender, *n* (%)			0.110
Female	17 (39)	5 (20)	
Male	27 (61)	20 (80)	
Age, years, median (range)	65 (27–89)	66 (29–84)	0.453
Age > 65 years, *n* (%)	21 (48)	14 (56)	0.509
BMI, kg/m^2^, median (range)	25 (18–40)	26 (19–34)	0.513
BMI > 30 kg/m^2^, *n* (%)	8 (19)	6 (24)	0.630
ASA physical status, *n* (%)			0.163
1	0 (0)	0 (0)	
2	21 (48)	11 (44)	
3	23 (52)	12 (48)	
4	0 (0)	2 (8)	
Comorbidities, *n* (%)			
Diabetes	8 (18)	4 (16)	1
Hypertension	19 (43)	10 (40)	0.797
Coronary heart disease	3 (7)	4 (16)	0.225
Pulmonary disease	1 (2)	3 (12)	0.132
Renal disease	4 (9)	2 (8)	1
Localization of primary, *n* (%)			0.423
Colon	22 (50)	15 (60)	
Rectum	22 (50)	10 (40)	
Synchronous CRLM, *n* (%)	19 (43)	13 (52)	0.480
Size of biggest CRLM > 50 mm, *n* (%)	7 (16)	3 (12)	0.657
Solitary CRLM, *n* (%)	16 (36)	19 (76)	0.002
CRLM in segments II, III, IVb, V, and VI, *n* (%)	42 (96)	21 (84)	0.180
CRLM in segments I, IVa, VII, and VIII, *n* (%)	25 (57)	11 (44)	0.306
Preoperative chemotherapy, *n* (%)	23 (52)	18 (72)	0.109
T stage of primary, *n* (%)			0.086
1	1 (2)	2 (8)	
2	8 (22)	0 (0)	
3	23 (62)	18 (75)	
4	5 (14)	4 (17)	
N stage of primary, *n* (%)			0.437
0	18 (49)	15 (63)	
1	12 (32)	4 (17)	
2	6 (16)	5 (21)	
3	1 (3)	0 (0)	
UICC stage of primary, *n* (%)			0.919
1	5 (12)	2 (8)	
2	8 (20)	6 (24)	
3	6 (15)	3 (12)	
4	22 (54)	14 (56)	
Tumor grading of primary, *n* (%)			0.412
G1	0 (0)	0 (0)	
G2	24 (89)	14 (78)	
G3	3 (11)	4 (22)	
Postoperative ICU stay, *n* (%)	40 (91)	17 (68)	0.022
Length of ICU stay, days, median (range)	1 (0–23)	2 (0–3)	0.283
Length of hospital stay, days, median (range)	9 (3–59)	5 (2–10)	<0.0001
90-day complications, *n* (%)	14 (32)	2 (8)	0.024
90-day major complications, *n* (%)	11 (25)	1 (4)	0.027
90-day mortality, *n* (%)	1 (2)	0 (0)	1
Anatomic resection, *n* (%)	26 (59)	1 (4)	<0.0001
Major resection, *n* (%)	22 (50)	1 (4)	<0.0001
Positive resection margins, *n* (%)	5 (11)	0 (0)	0.151
Resection margin width in R0 resected patients, mm, median (range)	4 (1–40)	3 (1–20)	0.519
Surgical technique, *n* (%)			0.002
right hepatectomy	11 (25)	1 (4)	
right hepatectomy and wedge resections or segmental resections	8 (18)	0 (0)	
left hepatectomy	1 (2)	0 (0)	
left hepatectomy and wedge resections or segmental resections	2 (4)	0 (0)	
left lateral hepatectomy	4 (9)	0 (0)	
wedge resection	13 (30)	17 (68)	
bisegmentectomy	5 (11)	7 (28)	
Need for transfusion, *n* (%)	5 (11)	3 (12)	1
Duration of operation, minutes, median (range)	290 (121–602)	125 (46–235)	<0.0001
Postoperative chemotherapy, *n* (%)	13 (30)	9 (36)	0.580

CRLM, colorectal liver metastases; BMI, body mass index; ASA, American Society of Anesthesiologists; UICC, Union internationale contre le cancer; ICU, intensive care unit.
